# Recent versus long-term maternal traumatic life events: Which one impacts prenatal attachment?

**DOI:** 10.1192/j.eurpsy.2021.556

**Published:** 2021-08-13

**Authors:** L.S. Meddouri, S. Bourgou, R. Fakhfakh, D. Bousnina, A. Triki, A. Belhadj

**Affiliations:** 1 Child And Adolescent, Mongi Slim Hospital, La Marsa, Tunisia; 2 Preventive Medecine, Aberahman Mami, ariana, Tunisia; 3 Preventive And Social Medecine, mother infant protection center, ezzouhour, Tunisia; 4 Gynecology And Obstetrics, Mongi Slim Hospital, La Marsa, Tunisia

**Keywords:** CTQ

## Abstract

**Introduction:**

Prenatal attachment is a strong predictor of post-natal attachment. Identifying factors influencing this bond is important, especially maternal history of stressful life-events.

**Objectives:**

Determine which type of maternal trauma impacts prenatal attachment.

**Methods:**

We conducted a transversal descriptive study in a first line clinical practice center and in an university gynecology-obstetrics department. We used Prenatal Attachment Inventory (PAI) to assess maternal-fetal attachment, the Childhood Trauma Questionnaire (CTQ) to evaluate maternal childhood stressful events and the Life-Threatening Events (LTE) to explore traumas during the past 6 months.

**Results:**

For the 125 pregnant women in our study, the mean age was 30 years and 5 months with 99,2% of them married. Mean gestational age was 33 weeks +1 day. PAI’s mean score was 55,58± 10,20; CTQ‘s mean score was 36,62 ± 9,53 revealing trauma in 28%. Women admitted being victims of IPV in 49,6% with almost the half (48,38%) being exposed to two or more forms of violence. Mean score for recent traumatic events in LTE was 1,87 with 65,2% being exposed to two or more life threatening event. A correlation between the total score of PAI and CTQ was found (p=0.021) particularly subscales of physical and emotional negligence of the CTQ (p=0.023 and p=0.006). We found no statistically significant correlation neither between PAI and IPV (p=0,453) nor between PAI and LTE (p= 0,360).

**Conclusions:**

Providing an appropriate training for health care providers can enable them to detect pregnancies at risk in order to refer them to trauma-informed mental health services.

